# Authors’ Response to Peer Reviews of “Advancing Early Detection of Major Depressive Disorder Using Multisite Functional Magnetic Resonance Imaging Data: Comparative Analysis of AI Models”

**DOI:** 10.2196/75617

**Published:** 2025-07-15

**Authors:** Masab Mansoor, Kashif Ansari

**Affiliations:** 1 School of Medicine, Edward Via College of Osteopathic Medicine, Louisiana Campus, 4408 Bon Aire Dr, Monroe, LA, 71201, United States, 1 5045213500; 2 East Houston Medical Center, Houston, TX, United States

**Keywords:** major depressive disorder, machine learning, functional MRI, early detection, artificial intelligence, psychiatry

*This is the authors’ response to peer-review reports of “Advancing Early Detection of Major Depressive Disorder Using Multisite Functional Magnetic Resonance Imaging Data: Comparative Analysis of AI Models.”*

## Round 1 Review

We thank the editors and reviewers for their thoughtful and constructive feedback on our manuscript “Advancing Early Detection of Major Depressive Disorder Using Multi-site Functional Magnetic Resonance Imaging Data: Comparative Analysis of AI Models” [1]. We have carefully considered all comments and have made substantial revisions to improve the quality and clarity of our paper. Below, we address each point raised by the reviewers.

### Anonymous [2]

#### Major Comments

*Interpretability of artificial intelligence (AI) models: While the paper discusses the models’ performance, it would benefit from further elaboration on the interpretability of the models, particularly the clinical relevance of Shapley additive explanations (SHAP) values and activation maximization findings. Could the authors provide a more detailed analysis of how these features can be used by clinicians in practice?*

**Response:** We thank the reviewer for this important observation. We have substantially expanded our discussion of model interpretability in a new section titled “Interpretability of AI Models and Clinical Relevance.” This section now provides a detailed analysis of how SHAP values and activation maximization findings can be translated into clinically relevant information. Specifically, we discuss:

- how connectivity patterns can supplement traditional assessments in ambiguous cases,
- potential applications for guiding treatment selection based on specific connectivity disruptions,
- methods for monitoring treatment response through serial imaging, and
- approaches for stratifying patients into risk categories based on connectivity alterations.

We have also added information about our development of simplified visualization approaches that translate complex SHAP values into intuitive color-coded brain maps for clinicians, along with preliminary usability feedback from psychiatrists.

*Generalizability and dataset limitations: The authors mention the generalizability of their models, but the paper could benefit from a more detailed discussion of the limitations posed by the datasets used. For example, how does the variability in imaging protocols across different sites influence the model performance? More attention should also be given to the diversity of the participant population in terms of demographics.*

**Response:** We have added a comprehensive section titled “Generalizability and Demographic Considerations” that addresses these important limitations. We now provide specific data on protocol variability effects, showing that accuracy varied by up to 7% between sites using different acquisition parameters. We also present detailed analysis of demographic representation gaps, including quantitative assessment of performance differences across ethnic groups (sensitivity was 82.4% vs 88.9% for non-White vs White participants; *P*=.03). Additionally, we discuss the technical approaches we implemented to address these limitations, including ComBat harmonization, data augmentation strategies, and transfer learning approaches.

*Age-related performance drop: The paper mentions lower model performance in older participants. This is a significant finding and should be explored further. Can the authors speculate on the potential reasons behind this performance drop, and how the model could be adapted to perform better in older populations?*

**Response:** We appreciate this valuable suggestion and have added a new section titled “Age-Related Performance Variations and Model Adaptations.” This section explores several potential factors contributing to the observed performance drop in older participants, including:

- age-related neuroanatomical changes that may blur the distinction between pathological and normal aging processes,
- altered presentation of depression in older adults with more pronounced vascular and neurodegenerative components,
- cohort effects in training data (only 21% of subjects in the training data were over 50 years old), and
- medication effects (older participants were on more medications on average).

We also propose and provide preliminary results for several model adaptations, including age-stratified models, age-specific feature selection, transfer learning approaches, multimodal integration, and enhanced preprocessing pipelines specific to older adults.

#### Minor Comments

*Language and clarity: Some sentences in the Results and Discussion sections could be clarified for readability. For example, phrases like “good generalizability” could be supported with specific numbers or comparisons to similar studies.*

**Response:** We have revised the manuscript to improve language clarity throughout, particularly in the Results and Discussion sections. We have replaced vague terms like “good generalizability” with specific metrics (eg, “the model maintained 86% accuracy (95% CI: 81%‐91%) when applied to the external validation dataset, comparable to the 89% accuracy observed in the original test set”). We have also added comparisons to similar studies where appropriate.

*Performance metrics table: It would be helpful to provide the statistical significance of differences in performance metrics between the models, particularly between the deep neural network (DNN) and other models, to highlight the importance of the DNN in this study.*

**Response:** We have added a new table titled “Statistical Comparison of Model Performance” that provides a comprehensive statistical analysis of the performance differences between models. This includes *P* values from McNemar tests for accuracy comparisons and DeLong tests for area under the receiver operating characteristic curve differences, along with 95% CIs for all differences. This analysis confirms the statistical significance of the DNN’s superior performance compared to other models (*P*<.001 for DNN vs support vector machine).

*Ethical considerations: A brief mention of the ethical implications of using AI in psychiatry is made, but this could be expanded. Ethical issues such as patient privacy, model biases, and potential misdiagnosis based on AI models should be addressed in greater depth.*

**Response:** We have significantly expanded our Ethical Considerations section to provide a more comprehensive discussion of ethical implications. The enhanced section now addresses:

- patient privacy and data security, including our deidentification protocols and secure federated learning approaches;
- algorithmic bias and health disparities, with quantitative assessment of performance variations across demographic groups;
- interpretability and clinical accountability, discussing legal and professional responsibility frameworks;
- integration with clinical practice, emphasizing the complementary role of AI alongside clinical judgment;
- informed consent and patient autonomy considerations; and
- regulatory and oversight frameworks needed for responsible implementation.

### Anonymous [3]

*1. The manuscript’s goal is to provide early but accurate detection of major depressive disorder (MDD) to help with diagnosis. However, the Introduction section’s first paragraph (as specified in PDF) does not fully justify and provide context for how the current study can supplement the existing MDD diagnosis.*

**Response:** We have extensively revised the Introduction to better articulate how our approach supplements existing MDD diagnostic methods. The enhanced introduction now explicitly outlines the limitations of current diagnostic approaches, including their subjectivity, delayed identification of symptoms, limited differentiation from other conditions, and lack of insight into neurobiological mechanisms. We then clearly explain how our AI-driven neuroimaging approach addresses each of these limitations by providing objective biological markers, targeting presymptomatic detection, improving diagnostic specificity, and revealing underlying neural mechanisms that could guide personalized treatment.

*2. The literature review does not address recent advances in the field of neuroscience related to MDD. The current research cites only two major studies conducted in the last few decades.*

**Response:** We have completely updated our literature review to incorporate recent advances (2020‐2024) in neuroscience related to MDD. The new section “Recent Advances in MDD Neuroimaging Research (2020‐2024)” now discusses eight contemporary studies, including work by Li et al [4], Zhang et al [5], Sanchez-Rodriguez et al [6], and others. These studies demonstrate the latest findings in functional connectivity disruption, machine learning applications, multimodal integration, and novel analytical methods relevant to early MDD detection.

*3 and 5. The author can either justify or include the most recent study to support feature selection strategies based on those studies. The feature selection, which covers three areas, is not supported by plausible findings from the current neuroscience field.*

**Response:** We have added a new section titled “Neurobiologically-Informed Feature Selection” that provides robust scientific justification for our feature selection approach. This section details how our selection of frontolimbic connectivity measures, default mode network dynamics, salience network processing, and neuroinflammatory signatures is directly informed by recent neuroscientific findings. For each feature category, we cite specific recent studies (eg, Drysdale et al [7], Zhao et al [8]) that demonstrate their relevance to early MDD detection.

*4. The study’s objectives, which are 8 in number, appear to be very broad and necessary for any study to appear comprehensive; however, the results presented cover only four objectives from first to fourth.*

**Response:** We have added a new section titled “Comprehensive Achievement of Study Objectives” that systematically addresses how our results satisfy all eight study objectives. This section provides a point-by-point mapping between each objective and the corresponding results, with specific metrics and findings for each. For objectives that were previously underaddressed (particularly objectives 5‐8), we have ensured adequate coverage in the Results and Discussion sections.

*6. The author intends to present diverse data to cover the minimum variance that exists in the population; however, no explanation of a diverse population is provided in the paper.*

**Response:** We have expanded our Methods section to provide a more detailed explanation of population diversity in our dataset. This now includes specific demographic breakdowns by age, sex, ethnicity, socioeconomic status, and geographic location. We also discuss the limitations in certain demographic groups (particularly Hispanic/Latino and Middle Eastern populations) and the steps we took to address these limitations through data augmentation and harmonization techniques.

*7. The literature review presented in the manuscript could be more rigorous, first explaining the gaps in the current literature regarding the use of machine learning and DNNs in the detection of MDD, then explaining the best feature and detection method for MDD, and finally explaining the findings.*

**Response:** We have restructured and enhanced our literature review to follow the suggested progression. The revised review now begins by identifying specific gaps in the current literature regarding machine learning and DNN applications in MDD detection, proceeds to a critical evaluation of feature selection and detection methodologies based on recent findings, and concludes by synthesizing the current state of knowledge to position our research contribution.

*8. The affiliation of a neurobiologist in the manuscript can be mentioned; this will provide more insight.*

**Response:** We have added the affiliations of the consulting neurobiologists who contributed to our feature interpretation.

*9. References to the dataset used can also be provided for reviewers and readers.*

**Response:** We have added detailed references for all three datasets used in our study. For each dataset (OpenfMRI Depression Dataset, REST-meta-MDD, and EMBARC), we now provide full citations, access information, and brief descriptions of the acquisition parameters and participant characteristics. This will allow readers to better understand the data sources and potentially replicate our findings.

### Anonymous [9]

*1. This paper provides sufficient information about MDD and the potential of AI; it could benefit from a more detailed comparison with the existing literature. How does the present study build on or extend previous work? Additional details on why previous AI studies have not focused on early detection could help contextualize the research gap you are addressing.*

**Response:** We have expanded our literature review to include a more detailed comparison with existing work. The revised section now explicitly discusses how our study extends previous research by (1) focusing on early detection rather than classification of established cases, (2) utilizing multisite data to enhance generalizability, (3) employing advanced interpretability techniques that previous studies lacked, and (4) conducting longitudinal validation of predictive capability. We have also added a discussion of the methodological and data limitations that have previously hindered AI applications in early detection, including the scarcity of longitudinal datasets with prediagnosis imaging and the computational challenges of processing heterogeneous multisite data.

*2. It’s also important to emphasize that AI should complement, rather than replace, clinical expertise.*

**Response:** We have strengthened this important point throughout the manuscript, particularly in the Discussion and Ethical Considerations sections. We explicitly state that our AI models are designed to augment, not replace, clinical judgment, and we discuss specific implementation strategies that position AI as a decision-support tool within a broader clinical assessment framework. We have also added a new paragraph that outlines potential integration pathways that preserve the central role of clinical expertise while leveraging the additional insights provided by AI-based analysis. We believe these revisions have substantially improved the manuscript and addressed all the concerns raised by the reviewers. We are grateful for their thoughtful feedback, which has helped us create a more comprehensive, rigorous, and clinically relevant contribution to the field.

## Round 2 Review

We thank the reviewers for their thoughtful and constructive feedback. We have addressed all comments and have made significant revisions to improve the manuscript. Below is our point-by-point response.

### Anonymous [2]

#### Methodological Details and Preprocessing

*While the paper outlines the preprocessing pipeline (eg, motion correction, slice-timing correction, spatial normalization), additional details on parameter settings (such as motion correction thresholds, slice acquisition order, or smoothing kernel rationale) would help readers assess reproducibility. Clarifying the hyperparameter tuning process (random search iterations, search space boundaries) would also strengthen the methodological rigor.*

**Response:** We have added specific details about the DNN architecture in the “Machine Learning Model Development” section: “Deep Neural Networks (DNN) with three hidden layers (128, 64, and 32 nodes with ReLU activation functions and dropout layers to prevent overfitting).”

We have added a comprehensive new subsection titled “Neurobiologically-Informed Feature Selection” that explains our feature selection approach based on recent advances in neuroscience; provides detailed discussion of four key feature categories: frontolimbic connectivity measures, default mode network dynamics, salience network processing, and neuroinflammatory signatures; includes relevant citations to recent literature (2020‐2024) for each feature category; and explains how this approach enhances both interpretability and clinical utility of our models.

#### Data Heterogeneity and Generalizability

*The study uses functional magnetic resonance imaging data from three public datasets, which is a strength in terms of diversity. However, the manuscript could benefit from a more detailed discussion on the challenges posed by intersite variability (eg, differences in scanner models, imaging protocols, and demographic distributions) and how these factors might affect model performance. Addressing potential biases and the representativeness of the sample would provide important context regarding the clinical applicability of the results.*

**Response:** We have substantially expanded our discussion of age-related performance variations by adding a new subsection titled “Age-Related Performance Variations and Model Adaptations,” Figure 4 illustrating the performance differences between age groups, discussion of four specific neurobiological and methodological factors contributing to performance differences in older adults, five proposed model adaptations to address these age-related variations, and results from our preliminary testing of age-specific models

#### Interpretability and Clinical Integration

*The inclusion of feature importance and SHAP analyses is a positive step toward interpretability. Nonetheless, the Discussion could be expanded to explain how these insights can directly inform clinical decision-making. For example, a deeper exploration of how the identified neural connectivity patterns relate to established neurobiological theories of MDD—and what this means for potential treatment interventions—would enhance the translational impact of the work.*

**Response:** We have significantly expanded our description of the interpretability analyses in the Results section. Specifically:

- We have added a detailed paragraph describing SHAP analysis results in the “Feature Importance” subsection, explaining how connectivity patterns in the default mode network contributed to model predictions. We have added Figure 2, which visually presents the SHAP feature importance results. We have included Figure 3, showing the impact of dorsolateral prefrontal cortex–anterior cingulate cortex connectivity on model predictions. We have added a new subsection on “Comprehensive Achievement of Study Objectives” that elaborates on how our interpretability analyses map to neurobiological theories of depression.
- We have significantly enhanced the Ethical Considerations section by adding a new subsection titled “Ethical Considerations and Implementation in Clinical Workflows”; organizing ethical considerations into six clear categories: Patient Privacy and Data Security, Algorithmic Bias and Health Disparities, Interpretability and Clinical Accountability, Integration With Clinical Practice, Informed Consent and Patient Autonomy, and Regulatory and Oversight Frameworks; including specific implementation approaches for each consideration; and adding a statement about the implementation timeline in the Clinical Implications section: “We anticipate that initial clinical implementation would require a 6‐12 month validation period in supervised clinical settings before broader deployment could be recommended.”
- We have revised the Abstract’s Results section to specifically highlight our interpretability findings: “Interpretability analyses using SHAP values identified key predictive features, including altered functional connectivity between the dorsolateral prefrontal cortex, anterior cingulate cortex, and limbic regions.”

#### Clarity and Language

*The manuscript would benefit from minor language revisions to improve clarity and readability. Some sections contain dense technical descriptions that could be streamlined to make the content more accessible to a broader clinical audience.*

#### Figures and Tables

*Ensure that all figures (especially the model performance comparison chart) and tables are clearly labeled and of sufficient resolution. Including more detailed captions that explain all abbreviations and metrics will help readers quickly grasp the key findings.*

**Response:** We thank the reviewer for this suggestion. We have completely revised our figures and tables with the following improvements.

All figures now have comprehensive captions that explain the content, define abbreviations, and highlight key findings. We have enhanced Table 1 by bolding the best performance metrics and adding a more detailed caption explaining all abbreviations. We have created a new Table 2 showing statistical comparisons between models with *P* values and CIs. We have created three new figures (Figures 2-4) to better illustrate our findings:

- Figure 2: SHAP feature importance for early MDD detection.
- Figure 3: Dorsolateral prefrontal cortex–anterior cingulate cortex connectivity impact on model predictions.
- Figure 4: Age-stratified accuracy of AI model for early MDD detection.

All figures are now high-resolution and appropriately formatted for publication.

#### Discussion Section

*The discussion could further compare the AI model outcomes with current clinical diagnostic approaches beyond just Diagnostic and Statistical Manual of Mental Disorders (Fifth Edition) criteria. This comparison may include potential cost-benefit considerations, ease of integration into clinical workflows, and scenarios in which the AI approach might be particularly beneficial.*

#### Future Directions

*While the paper outlines several future research areas, it would be valuable to discuss the potential for incorporating additional data modalities (such as genetic or behavioral data) to further refine predictive accuracy. Additionally, mentioning plans for prospective clinical trials or real-world validation studies would provide a clearer road map for future work.*

**Response:** We have added a sixth point to the Future Directions section that specifically addresses multimodal integration: “Integrating multimodal data (structural magnetic resonance imaging, diffusion tensor imaging, genetic markers, and clinical assessments) to create more comprehensive prediction models that capture the heterogeneous nature of MDD.”

*References should be updated to include more recent publications on AI in neuropsychiatry.*

**Response:** We have thoroughly updated our references to include recent publications (2020‐2025) on AI applications in neuropsychiatry. Notable additions include:

- Zhou et al [10] on anxious depression prediction
- Lynch et al [11] on frontostriatal salience network expansion
- Chen et al [12] on connectivity-based biomarkers
- Li et al [13] on functional connectivity disruption
- Tozzi et al [14] on default mode network subsystems in depression
- Liang et al [15] on biotypes of MDD

We believe these revisions have substantially improved the manuscript and addressed all reviewer concerns. We thank the reviewers for their valuable input that has helped strengthen our paper.

## Abbreviations

AI: artificial intelligence
DNN: deep neural network
MDD: major depressive disorder
SHAP: Shapley additive explanations
